# Vasogenic Edema Covering the Brain Surface in a Case of Severe Meningoencephalitis Due to Varicella-Zoster Virus Infection

**DOI:** 10.7759/cureus.63972

**Published:** 2024-07-06

**Authors:** Satoru Fujiwara, Kumiko Ando, Marie Tsunogae, Shigeki Arizono, Michi Kawamoto

**Affiliations:** 1 Department of Neurology, Kobe City Medical Center General Hospital, Kobe, JPN; 2 Department of Radiology, Kobe City Medical Center General Hospital, Kobe, JPN

**Keywords:** varicella zoster virus encephalitis, infection imaging, mri- magnetic resonance imaging, acute encephalitis, varicella-zoster virus

## Abstract

Meningoencephalitis caused by varicella-zoster virus (VZV) is a serious condition requiring prompt antiviral treatments, but magnetic resonance imaging (MRI) findings are often normal, limiting early diagnostic utility. We report a case of severe VZV-associated meningoencephalitis characterized by diffuse T2 hyperintense lesions covering the brain surface on MRI, presumed to be vasogenic edema. An immunocompetent 78-year-old Japanese woman presented with a disturbance of consciousness preceded by seven days of headache. On admission, she was in a semi-coma with intermittent convulsive seizures and had a localized skin rash with blisters on her back. Brain MRI showed diffuse T2 hyperintensity on the brain surface with an elevated apparent diffusion coefficient and the marked gadolinium-contrast enhancement of the pia-arachnoid membrane and vessel walls. Polymerase chain reaction using cerebrospinal fluid revealed the presence of VZV, and then she was diagnosed with VZV-associated meningoencephalitis. Treatment with acyclovir and corticosteroids was initiated, leading to some clinical improvement; however, the patient developed acute non-occlusive mesenteric ischemia and died on the 10th day of hospitalization. The characteristic MRI findings observed in our patient may be useful in considering the pathogenesis and early diagnosis of this rare entity.

## Introduction

Meningoencephalitis due to varicella-zoster virus (VZV) is an uncommon but serious condition that affects both immunocompromised and immunocompetent individuals [1,2]. Prompt intervention with antiviral drugs is of paramount importance, but magnetic resonance imaging (MRI) findings in this disease may often be normal, except for some reports of multiple infarcts, contrast enhancement of the meninges, or limbic encephalitis [3,4], and therefore not so useful for its early diagnosis.

Here, we report a case of severe VZV-associated meningoencephalitis characterized by T2 hyperintense lesions covering the brain surface. These lesions were presumed to be vasogenic edema, which may be an important finding in understanding the pathophysiology of this rare entity.

## Case presentation

A 78-year-old Japanese woman with no significant medical history was transferred due to a disturbance of consciousness. She developed a headache seven days before admission, followed by a rapid progression of impaired consciousness and confusion. On admission, she demonstrated semi-coma (Glasgow Coma Scale E1V1M4) with intermittent convulsive seizures that started from the right upper limb and generalized. There was a localized skin rash on the left back with some blisters. Laboratory evaluation showed no clue to impaired immune function (Table 1). Cerebrospinal fluid (CSF) analysis revealed pleocytosis (133 cells/μL, 34% lymphocytes) and highly elevated protein level (966 mg/dL).

**Table 1:** Laboratory values on admission Ig, immunoglobulin; AST, aspartate aminotransferase; ALT, alanine aminotransferase; BUN, blood urea nitrogen

Parameters	Patient’s results	Normal range
Total protein	7.5 g/dL	6.5–8.5 g/dL
Albumin	3.6 g/dL	3.9–4.9 g/dL
Globulin	3.9 g/dL	2.5–4.0 g/dL
IgG	1292 mg/dL	870–1700 mg/dL
IgM	74 mg/dL	35–220 mg/dL
IgA	318 mg/dL	110–410 mg/dL
AST	38 U/L	8–40 U/L
ALT	15 U/L	8–40 U/L
BUN	20.5 mg/dL	8.0–20.0 mg/dL
Creatinine	0.54 mg/dL	0.40–0.80 mg/dL
Glucose	236 mg/dL	70–110 mg/dL
Sodium	131 mEq/L	136–148 mEq/L
Potassium	3.4 mEq/L	3.5–5.3 mEq/L
White cell count	10.7×10⁹ /L	3.9–9.8×10⁹ /L
Neutrophils	88.5%	26–71%
Lymphocytes	6.0%	19–61%
Hemoglobin	16.4 g/dL	11.1–15.1 g/dL
Platelets	10.5×10⁴/µL	13.0–37.0×10⁴ /µL

**Figure 1:**
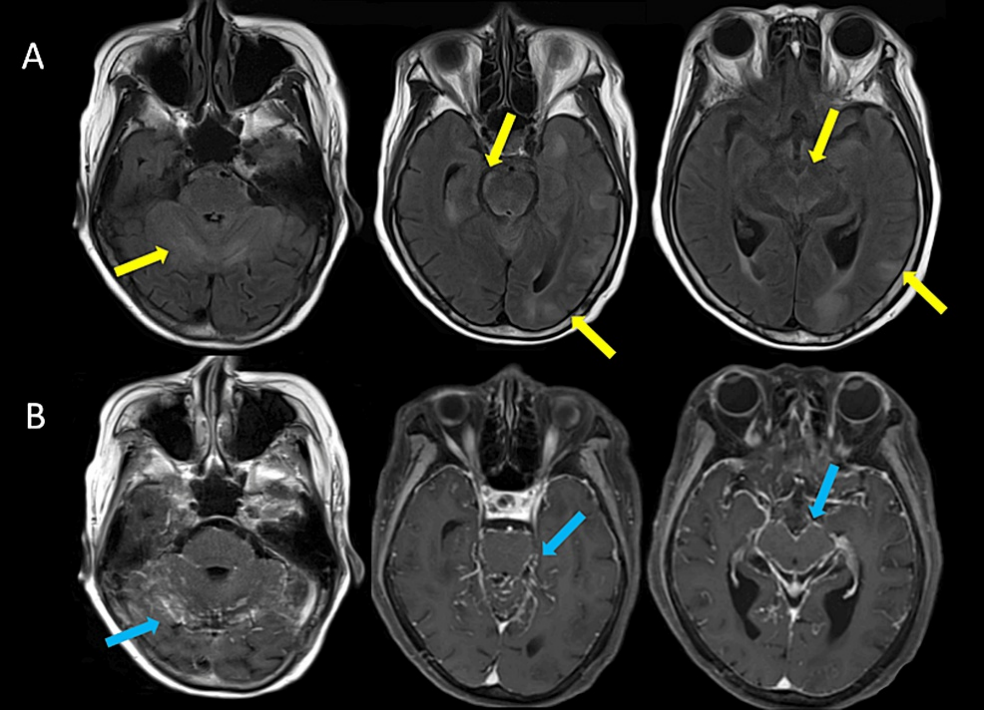
Brain MRI on admission A: FLAIR images showing showed multiple T2 high-intensity lesions on the surface of the cerebellum, brainstem, and cortex (yellow arrows); B: T1-weighted images with gadolinium enhancement (T1Gd) in which pia-arachnoid membrane and vessel walls of the brain surface showed a marked gadolinium-contrast enhancement effect (blue arrows). FLAIR: fluid-attenuated inversion recovery

Brain MRI revealed diffuse T2 hyperintensity covering the surface of the cerebellum, brainstem, and cortex (Figure 1A), with elevated apparent diffusion coefficient. The pia-arachnoid membrane of the brain surface showed marked gadolinium-contrast enhancement and the thickening of the blood vessel walls, which suggested severe inflammation (Figure 1B). Diffusion-weighted images showed no hyperintensities, while punctate hemorrhagic lesions were observed on susceptibility-weighted images.

Polymerase chain reaction (PCR) using CSF on the admission day showed > 10^8^ copies of VZV DNA per microliter. We then diagnosed her with VZV-associated encephalitis. Acyclovir (10 mg/kg every eight hours) and intravenous methylprednisolone pulse therapy (1000 mg for three days) were administered followed by oral prednisolone (40 mg/day). Clinical symptoms began to improve from the third day, with brief awakenings occurring one week later. T2 hyperintensity lesions on the brain surface had almost diminished on MRI on the sixth day. However, she developed acute non-occlusive mesenteric ischemia and unfortunately died on the 10th day of hospitalization. The autopsy was not performed as requested by the family.

## Discussion

VZV encephalitis is a rare condition that can occur in an immunocompetent individual, which is recognized as a vascular disorder that affects both small and large arteries [5]. The present case was highly characterized by T2 hyperintense lesions that appeared to cover the surface of the brain; to our knowledge, there have been no previous reports focusing on similar image findings.

The mechanism of this MRI finding remains unclear since no pathological evaluation was performed; however, this finding suggested that marked inflammation on leptomeninges or superficial vessels caused regional impaired perfusion, resulting in vasogenic edema on the brain surface. It has already been demonstrated by PCR that VZV invades the vessel wall [6], and it is known that VZV vasculopathy occurs in association with this pathology and is one of the risk factors for cerebral infarction. However, VZV vasculopathy generally has a chronic course [7], and why this case developed such a severe inflammation so soon after the onset of varicella is unknown. There might be a distinct pattern of central nervous system involvement of VZV in patients whose large vessels were vulnerable to this virus due to certain factors.

We believe that vasogenic edema covering the brain surface found in a patient with encephalitis suggests severe viral infection such as VZV, and prompt treatment be attempted.

## Conclusions

We described a case of VZV-associated meningoencephalitis presenting with the characteristic vasogenic edema covering the brain surface. This case highlights the need to consider severe VZV-associated meningoencephalitis and initiate antiviral treatment when similar MRI findings are observed.

The accumulation of more cases is crucial to elucidate the detailed pathophysiology and to determine the optimal treatment of this entity, which appears to be associated with significant inflammation.
